# Importance of individualized disaster preparedness for hospitalized or institutionalized patients: Lessons learned from the legal revisions made to the Basic Act on Disaster Management in Japan following the Fukushima nuclear disaster

**DOI:** 10.7189/jogh.11.03108

**Published:** 2021-10-02

**Authors:** Makoto Yoshida, Toyoaki Sawano, Yuki Senoo, Akihiko Ozaki, Yoshitaka Nishikawa, Tianchen Zhao, Hiroaki Saito, Yuzo Shimazu, Saori Nonaka, Yuji Moto, Chika Yamamoto, Masaharu Tsubokura

**Affiliations:** 1 Medical Governance Research Institute, Minato-ku, Tokyo, Japan; 2 Teikyo University School of Medicine, Itabahi-ku, Tokyo, Japan; 3 Department of Surgery, Jyoban Hospital of Tokiwa Foundation, Iwaki, Fukushima, Japan; 4 Department of Radiation Health Management, Fukushima Medical University School of Medicine, Fukushima, Fukushima, Japan; 5 Research Center for Community Medicine, Minamisoma Municipal General Hospital, Minamisoma, Fukushima, Japan; 6 Department of Breast Surgery, Jyoban Hospital of Tokiwa Foundation, Iwaki, Fukushima, Japan; 7 Department of Internal Medicine, Soma Central Hospital, Soma, Fukushima, Japan; 8 Department of Gastroenterology, Sendai Kousei Hospital, Sendai, Miyagi, Japan; 9 Southern Tohoku Research Institute for Neuroscience, Yatsuyamada, Koriyama, Fukushima, Japan; 10 Fukushima Red Cross Hospital, Fukushima, Fukushima, Japan

The global occurrence of natural and human-made disasters and emergencies is increasing. The fundamental goal of disaster preparedness, management, and recovery is to minimize damage to victims. However, disasters continue to affect billions of people and cause losses of lives and assets every year. Compared to other groups, vulnerable groups, particularly the elderly, people with disabilities, and institutionalized patients (living in hospitals and nursing and retirement facilities), experience heavier disaster burdens that prevent the normal provision of health services. To date, only limited legislation governing the provision and prosecution of specific mechanisms to protect the health of hospitalized/institutionalized patients during major disasters have been enacted. The establishment of a disaster-specific legal foundation to support the decision-making process and the necessary protective infrastructure, which should be developed in collaboration with medical professionals, as well as individuals with ethical and legal expertise, is a prerequisite to the protection of such populations during and after disasters. Japan, a disaster-prone country, has been continuously addressing and strengthening disaster risk management efforts, risk reduction frameworks, and relevant policies.

In Japan, the Basic Act on Disaster Management (BADM) is the foundation of all pre- and post-disaster management efforts. Recently, the focus of this law has shifted toward the protection of the health of vulnerable populations during disasters. The BADM was enacted in 1961 in response to the Isewan Typhoon (Typhoon Vera) in 1959, which killed 5089 people, and injured 38 921 individuals. The Japanese government has been amending the BADM almost annually to improve its disaster response. The first major amendment was made after the Great Hanshin (Osaka–Kobe) Earthquake (GHE) in 1995. This disaster’s impact on vulnerable populations was particularly severe, since more than half of the deaths occurred among the elderly. Based on the lessons learned from its experiences with the GHE, the Japanese government improved disaster risk management legislation by coordinating the expansion of the administrative function of the Emergency Response Headquarters and including steps to support vulnerable populations, such as the elderly and people with disabilities.

**Figure:**
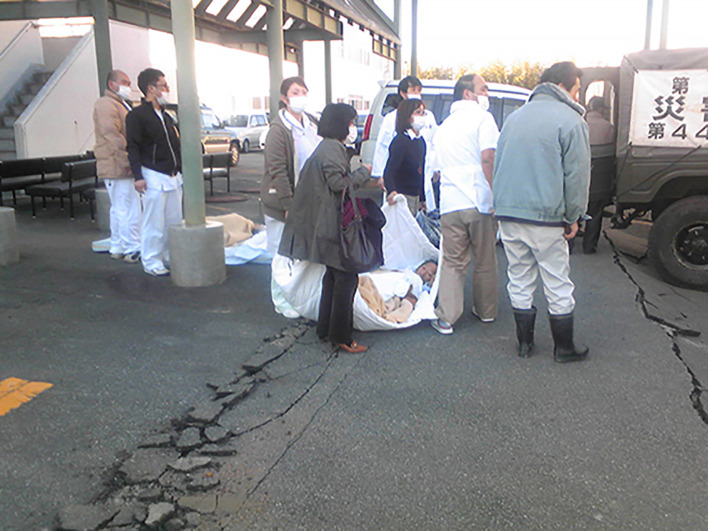
Photo: A scene of the emergency evacuation of patients at Futaba Kosei Hospital immediately after the Fukushima Daiichi nuclear power plant accident. The man on the bed sheet is a patient who is about to be evacuated in a Self-Defense Force vehicle (the photo was provided by Futaba Kosei Hospital, and used with permission).

The second major legal revision to the BADM was made in response to the Great East Japan Earthquake, which struck the northeastern part of mainland Japan and subsequently caused the Fukushima Daiichi Nuclear Power Plant (FDNPP) accident in 2011. As of March 2020, the disaster’s confirmed death toll was 15 899, among which 56.5% were people over 65 years of age [1]. Furthermore, a survey indicated that the mortality rate of people with disabilities was double (1.43%) that of the overall mortality rate of the region’s general population (0.78%), which highlights the inadequacy of disaster preparation efforts targeting vulnerable populations [2]. To enhance disaster preparedness for the vulnerable population, the government implemented a requirement for local governments to prepare a list of people who might require disaster evacuation assistance in its amendment of the BADM in 2013 [3].

Accordingly, despite responding reactively rather than proactively, the Japanese government implemented various disaster risk reduction measures targeting the vulnerable population; however, minimizing the impact of disasters on vulnerable populations, particularly hospitalized/institutionalized patients, remains challenging. In the past decade, Japan experienced disasters that forced scholars to recognize the difficulties involved in conducting evacuations for hospitalized/institutionalized patients. In this context, we discuss three events, as follows:

The first event is the evacuation of institutional residents carried out after the 2011 FDNPP accident. Two weeks after the FDNPP accident, a voluntary evacuation of nursing-home residents in Minamisoma City, Fukushima, was performed to mitigate radiation exposure. Studies on the risk of the evacuation of nursing home residents situated outside the compulsory evacuation zone revealed that 23% of the evacuated residents died within the first year of the evacuation [4], and showed a mortality risk approximately 1.82 times higher than the risk of non-evacuation [5]. A particularly catastrophic incident in the early phase of the Fukushima disaster was the evacuation of patients at Futaba Hospital, located 4.6 km away from the FDNPP. Among the 338 hospitalized patients, 39 (11.5%) died during the emergency evacuation, and most of the them were either bedridden or immobile without support [6]. Although the evacuation of nursing homes in Minamisoma City had a relatively long preparation period, studies reveal that the evacuation imposed severe physical and mental burdens on the nursing homes’ residents and caused an increase in long-term mortality [4,5]. On the other hand, at Futaba Hospital, inadequate care due to staff shortage, delayed evacuation due to communication failures, and prolonged evacuation-associated travel, in addition to changes in the hospital environment caused by infrastructure shutdown, might have contributed to the mass casualty.

The second event is Typhoon Lionrock, which occurred in late August 2016. It caused significant flooding, casualties, and property damage in Japan. Nine elderly nursing-home residents died as a result of the overflow of a nearby river in Iwaizumi Town, Iwate Prefecture [7]. It is believed that the municipality’s evacuation orders were delayed; furthermore, the facility itself did not have an evacuation manual for flooding nor a communication scheme to conduct an evacuation. All these resulted in a delay in rescue.

The torrential rain that occurred in the northern Kyushu region in July 2020 is the latest example of an unsuccessful evacuation. The heavy rainfall triggered several landslides in Kumamoto Prefecture and resulted in 65 deaths, among which approximately 70% were people over 70 years of age. The victims included the residents of a special nursing home in Kuma Village. As the result of a delayed evacuation, 14 died from flooding. Later, an investigation revealed that the nursing home staff had complied with the emergency evacuation plan and pre-disaster drills. However, human-power shortage and communication failures led to the spread of misinformation and made emergency evacuation plans insufficient, which delayed the evacuation. Finally, a nationwide survey conducted in response to the 2020 Kyushu flood revealed that many facilities for the elderly in Japan did not have any facility-specific evacuation plan [8]. Moreover, it revealed that some facilities were unable to prepare lists of people requiring disaster evacuation assistance since requests for personal health records were often declined by patients or their families [9].

These cases indicate that the limitation of legal instruction and the disaster management framework lies in the gap between relevant policy and its practice, and governmental action is generally reactive than proactive [10]. Based on the lessons learned from the emergency medical evacuation of hospitalized/institutionalized patients after the FDNPP accident, the BADM was amended to mandate local governments to establish alternative emergency evacuation sites and shelters in addition to the national government–designated emergency evacuation site. Furthermore, in response to the Iwate case in 2016, the central government amended the BADM-related act and mandated that facilities for elderly populations must establish an emergency evacuation plan and regularly conduct the disaster-specific evacuation drills.

Each disaster is unique and poses a wide range of health problems to victims depending on its location and setting. Therefore, there is a limit to establishing a disaster management plan with sufficient scope to cover the wide range of health issues experienced by hospitalized/institutionalized patients in various health care facilities after the disaster. Currently, in Japan, disaster preparedness efforts are experiencing several problems related to inadequate foresight, planning, and training, as indicated by the aforementioned cases. When a life-threatening disaster occurs, the best option is evacuation. However, for hospitalized/institutionalized patients, shelter-in-place may be an option depending on the risk balance. Since reports documenting the adverse effects of post-disaster evacuations of such vulnerable populations are currently limited, no specific strategy to maintain medical personnel and provide/maintain equipment and energy supplies onsite has been established for the shelter-in-place option. Furthermore, today, information sharing and responses of hospitals, national and local governments, and those engaged in onsite activities are poorly coordinated. In addition, it is necessary to establish official guidelines and laws to support and protect medical personnel responsible for determining emergency actions, such as choosing whether to evacuate or implement shelter-in-place based on limited onsite information.

To solve a series of disaster evacuation issues faced by hospitalized/institutionalized patients, it is necessary to comprehend the current disaster response and management situation. Subsequently, we discuss current issues and concerns, including those pertaining to local governments, onsite health care staff, and third-party organizations, based on existing evidence. A multi-level systems approach that encompasses the revision of laws, the establishment of a hospital-level or health care facility–level strategy, and researchers’ insights, is necessary to develop an effective disaster management plan for hospitalized/institutionalized patients. Moreover, we believe it particularly important to implement disaster management strategies directed toward encouraging decision-making authorities, such as the national and local governments, the Disaster Medical Assistance Team, municipal employees, medical professionals, and hospital directors, to engage in the planning process. An integrated disaster management model can improve the standardized disaster management strategy, since a study reported that different perceptions of responsibility among hospital executives and government officials potentially lead to different onsite actions [7]. It is important to understand the current situation of evacuation planning before conducting further research and realize that a few studies report the adverse effects of currently existing plans.
